# Case report: Thoracoscopic ablation for a patient with atrial fibrillation and persistent left superior vena cava

**DOI:** 10.3389/fcvm.2022.1096973

**Published:** 2023-01-18

**Authors:** Haozhong Liu, Tong Tan, Hailong Qiu, Jimei Chen, Jian Liu, Peijian Wei, Huiming Guo

**Affiliations:** ^1^Shantou University Medical College, Shantou, China; ^2^Department of Cardiovascular Surgery, Guangdong Cardiovascular Institute, Guangdong Provincial People's Hospital (Guangdong Academy of Medical Sciences), Southern Medical University, Guangzhou, China; ^3^Guangdong Provincial Key Laboratory of South China Structural Heart Disease, Guangzhou, China

**Keywords:** persistent left superior vena cava, atrial fibrillation, surgical ablation, thoracoscopic surgery, virtual reality

## Abstract

Persistent left superior vena cava (PLSVC) is a relatively rare congenital anomaly in the general population. It plays an important role in initiating and maintaining atrial fibrillation (AF) in some patients. Radiofrequency catheter ablation is the major treatment for patients with AF and PLSVC in most publications. Here, we reported a case of thoracoscopic ablation for a patient with atrial fibrillation and persistent left superior vena cava. After preprocedural simulation using virtual reality, we successfully completed box-lesion, ablation line from superior vena cava to inferior vena cava, left atrial appendage (LAA) excision, and PLSVC ablation. It provides a new perspective on surgical treatment for patients with AF and PLSVC.

## Introduction

During fetal development, the failure of obliteration of the left anterior cardinal vein and the left Cuvier's canal of the left superior vena cava in time would result in their continued existence after birth and the formation of PLSVC (1). Meanwhile, PLSVC has been previously reported to be a potential arrhythmogenic source of trigger or driver of AF (2, 3). Meanwhile, AF is associated with a five-fold risk of stroke (4). It has been reported that both surgical and catheter ablation of AF with different energy sources and lesion types have promising clinical outcomes (5–7). In this paper, we reported a case of thoracoscopic ablation for a patient with AF and PLSVC.

## Case presentation

**Figure 1:**
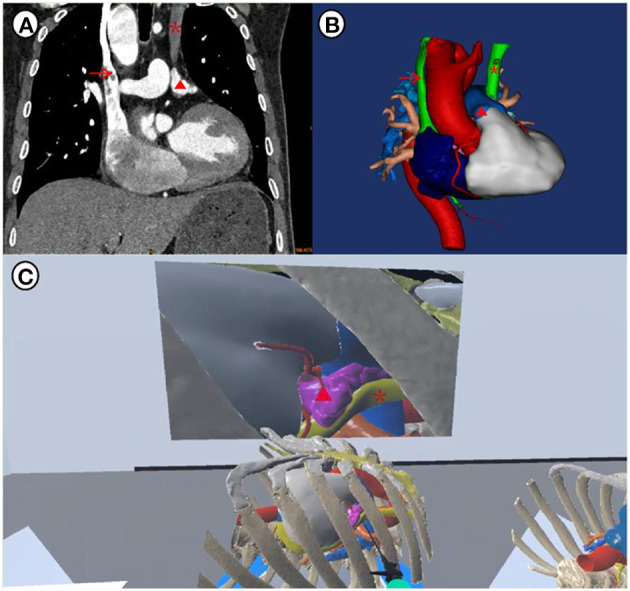
Preprocedural planning and simulation: **(A)** anatomical identification of PLSVC by CT; **(B)** three-dimensional reconstruction of the cardiac structures; **(C)** detection of the PLSVC and simulation of the thoracoscopic ablation using virtual reality tools. Surgeons' views can be obtained on this platform, and the 3D model can be zoomed in or out in a 360-degree version. The red triangle indicates left atrial appendage, the red arrow indicates right superior vena cava and the red asterisk indicates PLSVC.

A 61-year-old male patient was admitted to our hospital due to drug-refractory recurrent palpitations for 5 years. The electrocardiogram showed AF at 66 beats per minute. Echocardiography revealed the presence of PLSVC with an enlarged left atrium (57 mm) and right atrium (61 mm). Further computed tomography (CT) confirmed the identification of PLSVC (Figure 1A). Then three-dimensional reconstruction of cardiac structures was performed using the obtained CT images to visualize the anatomical features (Figure 1B). The PLSVC was shown to drain into the right atrium. Surgical ablation was required for this patient. However, the surgical approach is hard to determine since LAA Resection may cause injury to the PLSVC. Therefore, a preprocedural simulation of thoracoscopic ablation using virtual reality was performed and it verified the feasibility of the thoracoscopic approach (Figure 1C). So, we decided to perform bilateral two-port thoracoscopic ablation for this patient. Written informed consent was obtained from the patient.

**Figure 2:**
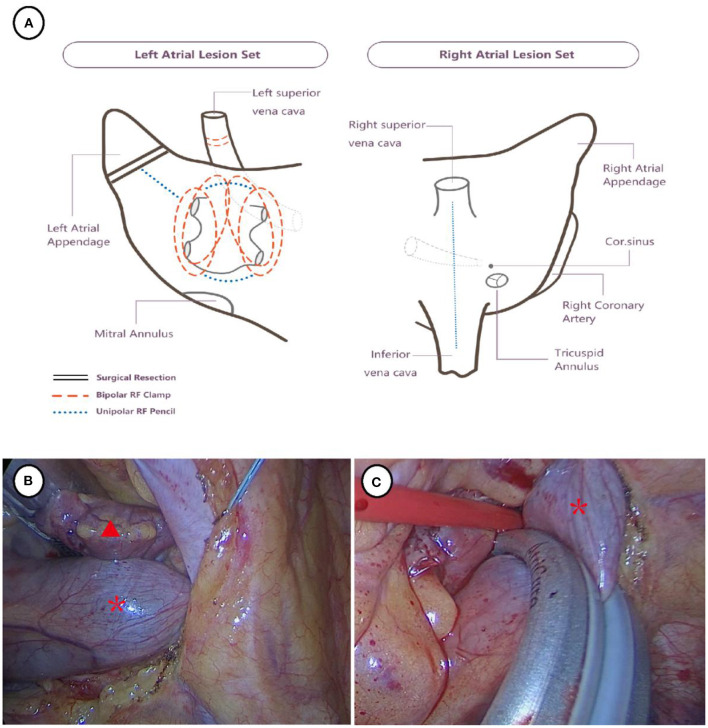
Surgical strategy **(A)** and thoracoscopic view of left atrial appendage excision and ablation of PLSVC **(B, C)**. The red triangle indicates left atrial appendage and the red asterisk indicates PLSVC.

Details of the procedure have been reported previously (8). The lesion set was shown in Figure 2A. Then the procedure was begun on the right side after left single lung ventilation. The pericardium was incised 2 cm anterior to the parallel phrenic nerve and suspended to expose the right atrium and pulmonary vein. The oblique and transverse sinuses were bluntly dissected, and the right pulmonary vein was ablated six times by inserting the AtriCure bipolar isolator (AtriCure. Inc., Ohio, USA) through the right inferior pulmonary vein. Subsequently, the AtriCure bipolar ablation pen was used to complete the ablation of the left roof line and floor line, the superior vena cava to the inferior vena cava line, and the coronary sinus. In the same manner, the thoracic cavity was entered from the left side except the assistant port was set third intercostal space. The left atrial appendage was completely resected with a stapler (Figure 2B). By steering clear of the PLSVC, the AtriCure bipolar isolator was inserted through the left inferior pulmonary vein, and the left pulmonary vein was then ablated six times (Figure 2C). The PLSVC was also carefully clamped and ablated in the case of blood pressure stabilization. The intraoperative electrocardiogram revealed sinus rhythm. At the 12-month follow-up, the electrocardiogram showed sinus and rhythmic rhythm. No major adverse events (bleeding and thromboembolic events) were reported.

## Discussion

PLSVC is a relatively rare congenital anomaly, but it is one of the most common systemic venous anomalies in the thoracic cavity and has been reported to occur in 0.3–2% of the otherwise normal population and up to 10% of patients with congenital heart disease (9, 10). The current literature suggests that it is an incidental finding that often occurs with central venous catheters through the internal jugular vein or the subclavian vein. There are four anatomic types of PLSVC: PLSVC with atresia of the right superior vena cava (type 1); PLSVC draining into the right atrium with (type 2A) or without (type 2B) an anastomosis with right superior vena cava; and PLSVC draining into the left atrium (type 3) (11). Type 1 and 2 PLSVC have a higher frequency, which therein passes through the coronary sinus into the right atrium and therefore do not have significant hemodynamic consequences and in most cases manifest asymptomatically (12). However, PLSVC may be the arrhythmic source of persistent AF (2, 3), which is reasonable to apply surgical, catheter, or hybrid ablation (13). In this case, the patient preferred a minimally invasive surgical approach. Most AF patients with PLSVC were reported to undergo radiofrequency ablation, but data were limited and the long-term outcomes of freedom from AF were awaiting. During the thoracoscopic surgery, we were also allowed to perform left atrial appendage excision, which would further decrease the further adverse events like stroke. Besides, PLSVC can distort the normal anatomy of the atrium, leading to a higher risk of procedure complications, such as bleeding, and cardiac tamponade (3). On the contrary, thoracoscopic ablation offered a wide field of cardiac structures. Overall, surgical ablation through a double port thoracoscopic approach might be a more efficient and safer strategy for AF patients with PLSVC.

In this case of thoracoscopic ablation for a patient with AF and PLSVC, better visualization and surgical simulation were achieved by three-dimensional reconstruction and virtual reality technique. However, the first dilemma was the feasibility of thoracoscopic surgical ablation. The PLSVC sterically blocked our insulator from reaching the pulmonary veins. By measuring the diameter of the PLSVC and observing its relationship with surrounding structures, the diagnosis of a type 2B PLSVC was reconfirmed, and we concluded that the PLSVC could be bypassed without incisional enlargement or cardiopulmonary bypass. During the surgical simulation, we assumed the operation hole was set at the 4th rib, and the virtual visual field of thoracoscopy showed that both the PLSVC and the left atrial appendage were exposed within the operating range. Lu et al. (14) had a similar experience of deciding on a treatment strategy by using the 3D acquisition technique to understand the vascular anatomy of the PLSVC.

In addition, when clamping the PLSVC for ablation, the venous volume return to the heart might suddenly decrease, leading to a dipping in blood pressure. In such cases, cooperation between the surgeon and the anesthetist is required. The PLSVC should be relieved till to the hemodynamics return stable. Volume management and vasoactive agents were required when necessary. To achieve PLSVC ablation, we preferred performing more times instead of each long-time scale. There might be an extremely rare condition which only the PLSVC is complicated by atrial fibrillation while the right superior vena cava (RSVC) is absent. In such a scenario, decision to ablate the LSVC depends on the adjacent structures and the diameter of the left superior cavity. On the one hand, if the cavity of LSVC is too small, it should not be ablated because the risk of LSVC stenosis is too high. On the contrary, if the lumen of LSVC was large enough and easy to separate from adjacent tissue, we thought it would be reasonable to perform ablation.

In this case, we confirmed that thoracoscopic ablation can be a feasible surgical option for patients with AF and PLSVC.

## Data availability statement

The original contributions presented in the study are included in the article/supplementary material, further inquiries can be directed to the corresponding authors.

## Ethics statement

The studies involving human participants were reviewed and approved by Ethics Committee of Guangdong Provincial People's Hospital, Guangdong Academy of Medical Sciences. The patients/participants provided their written informed consent to participate in this study. Written informed consent was obtained from the individual(s) for the publication of any potentially identifiable images or data included in this article.

## Author contributions

HL and TT contributed to the study design and manuscript drafting. HQ contributed to data acquisition. JC, PW, and HG contributed greatly to the revision of the manuscript. JL approved the submission of the final version. All authors contributed to the article and approved the submitted version.
